# Age-Specific Trends of Invasive Cervical Cancer Incidence in British Columbia, Canada, 1971–2017

**DOI:** 10.3390/curroncol30080557

**Published:** 2023-08-18

**Authors:** Nivedha Raveinthiranathan, Jonathan Simkin, Robine Donken, Gina Ogilvie, Laurie Smith, Dirk Van Niekerk, Marette Lee, Ryan R. Woods

**Affiliations:** 1 Faculty of Health Sciences, Simon Fraser University, Burnaby, BC V5A 1S6, Canada; 2 Cancer Control Research, BC Cancer, Vancouver, BC V5Z 1L3, Canada; 3 School of Population and Public Health, University of British Columbia, Vancouver, BC V6T 1Z3, Canada; 4 Women’s Health Research Institute, BC Women’s Hospital and Health Service, Vancouver, BC V6H 3N1, Canada; 5 Vaccine Evaluation Center, BC Children’s Hospital Research Institute, Vancouver, BC V5Z 4H4, Canada; 6 Cervical Cancer Screening Program, BC Cancer, Vancouver, BC V5Z 1G1, Canada

**Keywords:** cervical cancer, cancer incidence, population surveillance, cancer control and prevention, elimination, Canada

## Abstract

This study examined invasive cervical cancer (ICC) incidence trends in British Columbia (BC) by age and stage-at-diagnosis relative to World Health Organization ICC elimination targets (4 per 100,000 persons). Incident ICC cases (1971–2017) were obtained from the BC Cancer Registry. Annual age-standardized incidence rates (ASIRs) per 100,000 persons were generated using the direct method. ASIRs were examined among all ages 15+ years and eight age groups using Joinpoint Regression with the Canadian 2011 standard population. Standardized rate ratios (SRRs) compared stage II–IV (late) versus stage I (early) ASIRs by age (2010–2017). ICC ASIRs did not reach the elimination target. ASIRs declined from 18.88 to 7.08 per 100,000 persons (1971–2017). Stronger declines were observed among ages 45+ years, with the largest decline among ages 70–79 years (AAPC = −3.2%, 95% CI = −3.9% to −2.6%). Among ages 25–69 years, varying levels of attenuation in declining trends and stabilization were observed since the 1980s. SRRs indicated higher rates of late-stage ICC among ages 55+ years (SRR−55–69 years = 1.34, 95% CI = 1.08–1.71). Overall, ICC incidence declined in BC since 1971 but did not reach the elimination target. The pace of decline varied across age groups and increased with age. Continued efforts are needed to progress cervical cancer elimination among all age groups.

## 1. Introduction

Globally, cervical cancer remains the fourth most common cancer among women [1]. In Canada, 1450 new cervical cancer diagnoses and 380 cervical cancer deaths were projected to occur in 2022 [2]. Persistent infection with oncogenic genotypes of the human papillomavirus (HPV) is considered a necessary cause of cervical cancer [3]. Cervical cancer morbidity and mortality are significantly reduced via primary prevention through vaccination, secondary prevention through screening and early detection, and tertiary prevention through effective management [4].

Significant reductions in cervical cancer incidence have been observed in Canada and in different populations globally, largely attributed to the introduction of cytology-based screening programs [5,6,7,8,9,10,11,12,13,14,15,16]. Reductions in cervical cancer incidence may not have been equally distributed across age groups. In Canada, studies have found that declines in incidence generally increase with age, with older women ages 45 years and above experiencing the largest reductions [6,8,10]. Similar trends have been reported in other countries with comparable cancer surveillance systems including the United States (US) and some countries in northern Europe [11,14,17,18].

Further, a recent Canadian analysis showed that, while most cervical cancers were diagnosed at Stage I, 12% were still diagnosed at Stage IV with the proportion of Stage IV diagnoses increasing with age [19]. In the US, localized cervical cancers are associated with a 5-year net survival of 83%, while those with distant metastases are associated with 17% survival [20]. 

The World Health Organization (WHO) has called for accelerating efforts to eliminate cervical cancer globally, with an elimination target of 4 cases per 100,000 persons [21]. With Canada among the leading countries to meet this goal [22], maximizing the reach and impact of cervical cancer prevention and screening efforts remains critical. The Canadian Partnership Against Cancer, an independent, federally funded organization tasked with accelerating action on cancer control, has outlined a specific action plan for elimination in Canada [23]. Pathways to elimination include improving uptake of HPV vaccines, implementing HPV-based screening technologies, improving screening participation and addressing inequities in access and availability of services to underserved populations [23,24].

While national trends in cervical cancer incidence have been well characterized [5,6,8,9,10,25], analyses of provincial trends by age and stage are incomplete. In addition to examining cervical cancer incidence trends in a large provincial population with an advanced screening system, we aim to update British Columbia (BC)-specific data on trends to identify key gaps and groups to support progress toward the WHO call for cervical cancer elimination. The purpose of this study was to use population-based data from the BC Cancer Registry to examine incidence trends of invasive cervical cancer by age and stage-at-diagnosis over a 47-year period in relation to cervical cancer elimination targets.

## 2. Materials and Methods

There were approximately 2.6 million people with a cervix residing in BC in 2020, including women, two-spirit, transgender and non-binary people [26]. The first organized cervical cancer screening program in Canada was established in BC in 1960 and continues to be operated by BC Cancer [27]. Under current health policy, routine cervical cancer screening using cytology testing is publicly funded for women aged 25–69 years, as per national recommendations [27,28].

Registered cases of invasive cervical cancer occurring between 1971 and 2017 were derived from the BC Cancer Registry (BCCR), a population-based provincial registry that contains diagnostic and demographic data for all incident cancers diagnosed among BC residents. It is a gold-certified cancer registry as per the North American Association of Central Cancer Registries certification criteria and estimated to capture over 95% of all reportable cancer cases in BC [29]. Cancers in the BCCR are staged using the seventh edition of the TNM staging system. Provincial, national, and global population estimates were derived from publicly available datasets from Statistics Canada and the United Nations [30,31].

The outcome of interest was the incidence of primary invasive cervical cancer, identified as site code C53 according to the International Classification of Diseases for Oncology, Third Edition [32]. Covariates included age and stage at diagnosis.

Age-standardized incidence rates (ASIRs) per 100,000 persons were generated using the direct method. Following recommendations for country-specific analyses to standardize to both local and global age structures [33,34], incidence rates were standardized to the Canadian 2011 population and the 2015 world female population. The latter was recommended for global comparisons of incidence and in relation to the WHO cervical cancer elimination threshold [33,34], which is 4 cases per 100,000 persons [4].

Trends in ASIRs among all ages 15+ years and within eight age groupings were examined. Age groups were selected to reflect current screening guidelines in BC [27]: 15 to 19 years, 20 to 24 years, 25 to 34 years, 35 to 44 years, 45 to 54 years, 55 to 69 years, 70 to 79 years and 80+ years. Ages < 15 years were excluded due to zero cases of cervical cancer across the study period. Trends in annual ASIRs were analyzed using the Joinpoint Regression Program, version 4.8.0.1, developed by the National Cancer Institute [35]. A *p*-value < 0.05 on a permutation test was used to identify statistically significant joinpoints [36]. We report two key measures of the trend with corresponding 95% confidence intervals (CIs): (1) the annual percentage change (APC) in each individual trend segment and (2) the average annual percentage change (AAPC) [36]. Modeling parameters used for Joinpoint analysis follow those previously described in national cancer surveillance reports [37]. Joinpoint is able to estimate trends only when all years within a given period have at least one incident count among all age strata. Thus, we could not quantify trends among age groups 15–19 and 20–24 years, where there were several years with zero cases. The statistical computing language R [38], program R Studio [39], and R package ‘epitools’ [40] were used to calculate ASIRs among these age groups with zero counts. 

Stage-specific analysis was limited to diagnosis years 2010 to 2017 as population-based stage data were only available in the cancer registry from 2010. This period was aggregated due to small annual stage-specific and age-specific case counts. Stage at diagnosis was aggregated into early (Stage I) and late (Stage II–IV) stage. Cases staged as “unknown” were excluded from this analysis (*N* = 77). The standardized rate ratio (SRR) for late- versus early-stage cervical cancer was calculated for each age group with corresponding 95% confidence intervals (CIs) [41]. 

## 3. Results

### 3.1. Incidence Trends among Ages 15+ Years, 1971–2017

**Figure 1:**
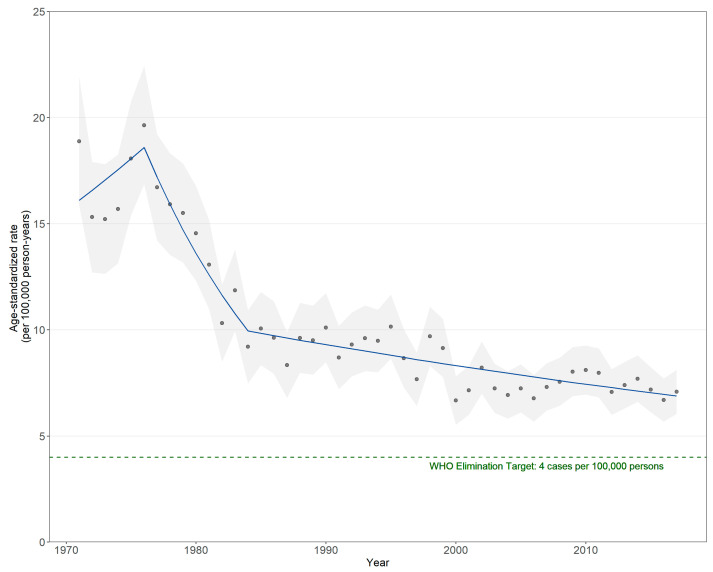
Age-standardized incidence rates of invasive cervical cancer and 95% confidence interval by year and Joinpoint trend lines for ages 15+ years, 1971–2017. Calculated with the Canadian 2011 Census population standard. The dark-blue lines indicate trend lines generated by Joinpoint trend analysis software. Gray-shaded areas indicate 95% confidence intervals.

**Figure A1:**
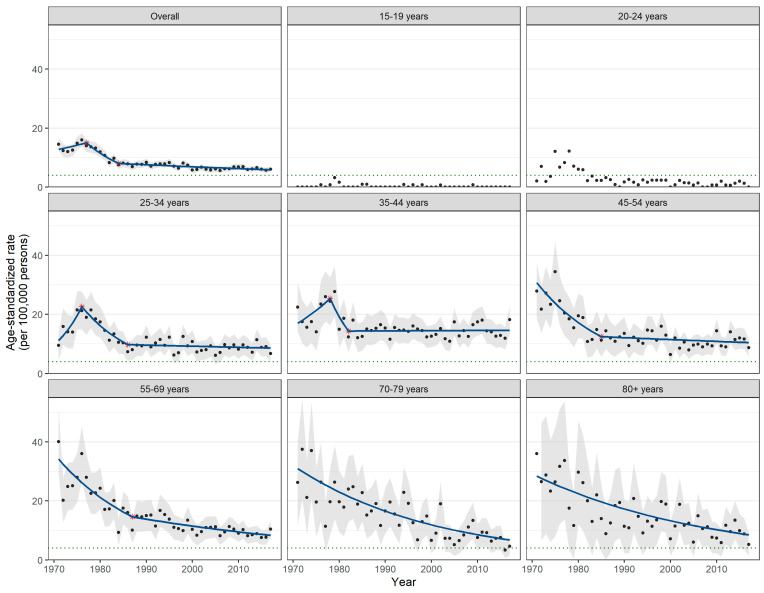
Overall and age-specific annual ASIRs of invasive cervical cancer in British Columbia, 1971–2017. ASIRs = age-standardized incidence rates. Calculated using the 2015 World Female Population Standard. Dark-blue lines indicate trend lines generated by Joinpoint trend analysis software. Red asterisks indicate statistically significant joinpoints in trends. Gray-shaded areas indicate 95% confidence intervals. Trends could not be computed for ages 15–19 and 20–24 years due to zero case counts in multiple years.

Between 1971 and 2017, the annual ASIR declined from 18.88 to 7.08 cases per 100,000 persons, corresponding to an AAPC of −1.8% (95% CI −2.7% to −1.0%) (Figure 1 and Appendix A, Table A1 and Table A2). Overall incidence rates did not reach the WHO elimination target of 4 cases per 100,000 persons when standardized to the 2011 Canadian population. In the most recent 10-year period from 2008 to 2017, some attenuation was observed in the overall trend with an AAPC of −1.1% (95% CI −1.4% to −0.8%). Incidence rates first began to decline in 1976 until 1984, corresponding to an APC of −7.5% (95% CI −10.6% to −4.3%); the rate of decline then reduced to an APC of −1.1% (95% CI −1.4% to −0.8%) for the rest of the study period. The WHO elimination target was not reached when overall incidence rates were standardized to the 2015 world female population (Appendix A, Figure A1, Table A1 and Table A3).

### 3.2. Incidence Trends by Age

**Figure 2:**
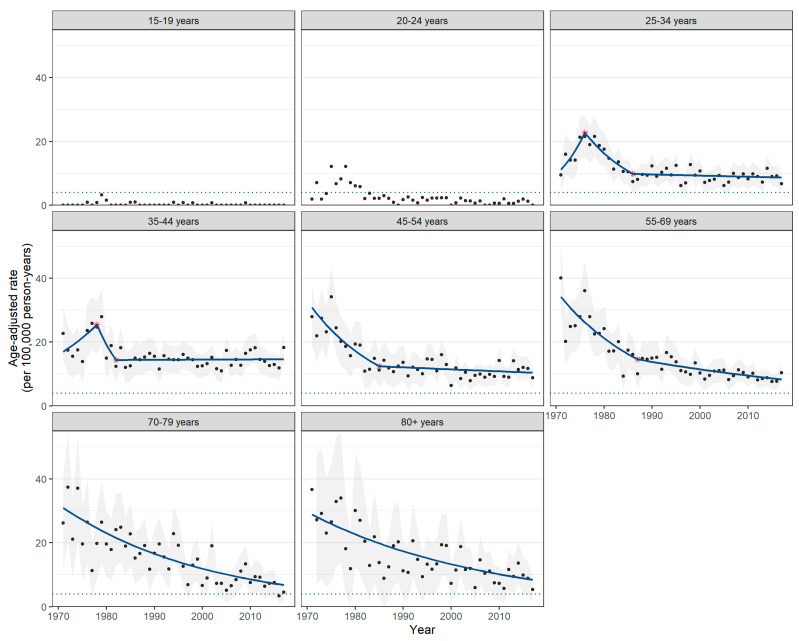
Age-standardized incidence rates of invasive cervical cancer, 95% confidence intervals, and Joinpoint trend lines by year and age group, 1971–2017. Calculated with the Canadian 2011 Census population standard. Dark-blue lines indicate trend lines generated by Joinpoint trend analysis software. Red asterisks indicate statistically significant joinpoints in trends. The dashed green line indicates the WHO cervical cancer elimination target (4 cases per 100,000 persons). Gray-shaded areas indicate 95% confidence intervals. Trends could not be computed for ages 15–19 and 20–24 years due to zero case counts in multiple years.

Annual age-specific ASIRs and incidence trends over the 1971–2017 period are presented in Figure 2. The corresponding APC and AAPC values are summarized in Appendix A, Table A2. Incidence trends for age groups 15–19 and 20–24 years could not be modeled using Joinpoint due to zero case counts in multiple years. Annual ASIRs in the youngest age groups remained below the WHO elimination target for most of the study period (Figure 2).

For age groups 25 years and above, the incidence of cervical cancer declined across the study period but at varying rates. Generally, greater reductions in incidence were seen for older age groups. For example, ages 70–79 years had the largest reduction in incidence with an AAPC of −3.2% (95% CI −3.9% to −2.6%). In contrast, the rate of decline among ages 35–44 years corresponded to a non-significant AAPC value of −0.3% (95% CI −2.6% to 2.0%) across the study period.

The two oldest age groups showed a consistent declining trend with no significant change during the study period. In contrast, incidence trends among younger age groups showed varying levels of attenuation or stabilization, typically starting in the 1980s. For example, ASIRs among ages 25–34 years initially declined with an APC of −8.0% (95% CI −11.8% to −4.1%) between 1976 and 1986, but then reduced to −0.4% (95% CI −1.1% to 0.3%) until 2017. Similar attenuation was seen among ages 45–54 years and 55–69 years.

### 3.3. Incidence Trends by Age and Stage

**Figure 3:**
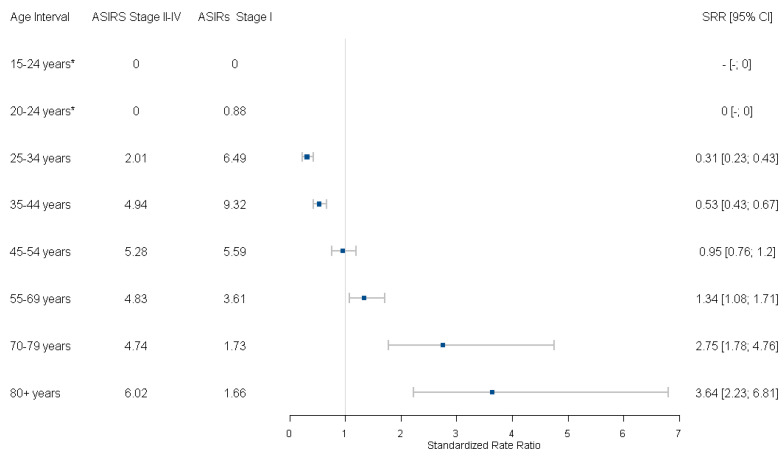
Age-standardized incidence rates and standardized incidence ratios of invasive cervical cancer by stage at diagnosis group (stage II–IV versus stage I) and age groups, 2010–2017. * Age groups had zero case counts of early or late stage cancers. ASIR = age-standardized incidence rate; SRR = standardized incidence ratio; CI = confidence intervals.

The stage- and age-specific ASIRs for the 2010–2017 period are summarized in Figure 3, along with SRRs of late- to early-stage cervical cancer. The ASIR of early-stage (Stage I) and late-stage (Stage II to IV) cancers during this period was 3.89 cases per 100,000 persons and 3.14 cases per 100,000 persons among all age groups combined (SRR = 0.81; 95% CI 0.73 to 0.90), respectively.

Lower rates of late-stage cancers relative to the early stage were observed among younger age groups between 25 and 44 years, with the lowest SRR corresponding to ages 25–34 years (SRR = 0.31; 95% CI 0.23 to 0.43). Rates of early- and late-stage cancers were similar among the age group 45–54 years (SRR = 0.95; 95% CI 0.76 to 1.20). A reversal of the pattern was observed in older age groups with higher rates of late-stage cancers relative to early-stage cancers, with the highest SRR corresponding to ages 80+ (SRR = 3.64; 95% CI 2.23 to 6.81). 

## 4. Discussion

Invasive cervical cancer incidence (ICC) rates have declined among those aged 15 or older and across most age groups in BC since 1971. However, the pace of decline varied among age groups. Trends in incidence among those aged 70 years and above showed a consistent strong decline over the study period. Other than those aged less than 25 years, the ICC rate did not reach the WHO elimination target over the study period when standardized to either the 2011 Canadian or the 2015 World Female Population standards. Among those aged 25–69 years, varying levels of attenuation in declining trends and stabilization were observed since the 1980s, particularly among the younger groups. Further, women aged 25–44 years showed higher rates of Stage I cancers relative to later-stage cancers, whereas those aged 55 years and older showed higher rates of cancers diagnosed at later stages.

High-income countries with long-established and widespread prevention, screening, and treatment modalities are positioned to rapidly reach the cervical cancer elimination target within the coming decades [42]. Importantly, as a persistent infection with an oncogenic HPV type is necessary for the development of nearly all cervical cancer, receipt of HPV vaccination prior to onset of sexual activity is highly effective at preventing infection, as well as cervical pre-cancer and cancer [43]. In Australia, it is predicted that, with sustained coverage of HPV vaccination and screening at current levels, elimination is likely to be achieved by 2028 [44]. The US is on track to eliminate cervical cancer by 2038–2046, with possibly an even shorter time to elimination by increasing screening coverage [34]. In BC, HPV vaccination is provided free of charge to girls and boys in grade six through a school-based immunization program. However, provincial vaccine uptake rates in 2019 were 66.1% and 63.5% for females and males, respectively [45], which is well below the WHO vaccination target of 90% needed for elimination.

Our findings are consistent with previously reported reductions in cervical cancer incidence trends in Canada and other high-income countries [6,8,9,10,11,12,13,14,46,47]. To date, reductions in cervical cancer incidence have been attributed to cytology-based screening programs. 

One Canadian study reported a decrease in national incidence rates from 22.3 to 9.4 cases per 100,000 women between 1972 and 2006, corresponding to a 58% reduction [6]. Declines were reflected across all Canadian provinces but at varying rates [9]. While significant declines have been observed in Canada, attenuation in declining trends have been reported in recent periods [6,10]. Potential factors related to attenuation include changes in behavioral risk factors, greater immigration from countries with higher cervical cancer rates, and gaps in screening for immigrant, low-income, rural, and Indigenous women who are less likely to be screened [24,48,49]. While further decreases in Canada can be expected as vaccinated cohorts reach the ages when cervical cancer is commonly diagnosed [10], this effect is likely not apparent in this study’s results as the oldest cohort eligible for the vaccination program in BC was born in 1994 (they would have only been 23 years old in 2017).

In the US, declining rates stabilized in more recent periods (2011–2015), driven by trends among subpopulations such as white women aged 30–59 years [11]. In the United Kingdom, large declines in cervical cancer incidence were seen following the implementation of organized cervical screening in 1988 [14]. Despite declining trends, the rate of change was lower among Northern European countries, and stabilization of trends was noted in more recent periods [13]. Studies suggest that this is related to changes across successive birth cohorts, with one study reporting increases in ICC risk among successive cohorts of women born in 1940–1950 and thereafter in nearly all European countries while no substantial changes were seen in North America [13].

Our findings are consistent with studies that reported declines in most age groups but greater reductions among older age groups. In Canada, the largest reductions in incidence rates were observed among women aged 45 years and above (57–69%), while more modest reductions (26–36%) were observed in women aged 25–39 years between 1972 and 2006 [6]. Data up to 2015 indicate a continuation of these trends, with APC values progressively decreasing from −0.61 to −8.80 in age groups above 40 years, whereas the 30–39 year age group had a non-significant APC of −0.33 (CI −1.16 to 0.50) [10].

Several studies examined age effects by histological type of cervical cancer. Approximately 75% of all cervical cancers are squamous cell carcinomas (SCCs), while the remainder comprise adenocarcinomas (ACs) and other types of cervical cancer including adenosquamous carcinomas. Overall patterns in cervical cancer incidence are thought to be driven by declines in SCC, the type most impacted by cervical screening [50]. A Canadian study showed that SCC incidence rates declined among all ages between 1970 and 1996, with larger reductions for older women [8]. In contrast, AC and adenosquamous carcinoma incidence rates have steadily increased for women 20–49 years of age [8]. A US study concluded that overall SCC rates have continued to decline in all racial/ethnic groups except for non-Hispanic whites, for whom rates have stabilized since 2010 [11]. Among non-Hispanic whites, the overall trend was driven by stable trends in women younger than 50 years and slow declines among those aged 50–59 years [11]. In contrast, AC trends increased in non-Hispanic whites during 2002–2015 largely due to increases in ages 40–59 years [11]. Studies in Northern European countries also report greater stabilizing SCC trends and increasing incidence of AC among young women [14,15,16].

Some studies suggested a possible cohort effect driving age-specific trends. In the US, increased risk of AC is related to changing sexual behaviors and increased use of oral contraceptives in successive birth cohorts of women since the 1960s [11,18]. Further, the lesser sensitivity of cytology testing for AC than SCC was cited as a possible driver of increasing AC trends. In many northern European countries, differing trends of SCC and AC among younger women were noted across birth cohorts and attributed to generational changes in sexual behavior, such as sexual debut at earlier ages and a higher number of sexual partners [14,16,46,47]. Increasing AC trends among younger women were attributed to this cohort effect, while the slower pace of decline in SCC in was related to competing cohort-specific increases in risk of cervical SCC, counteracting the period-specific effects of screening [14,16,17,46,47]. 

Our findings support previous evidence that incidence in the youngest age groups (15–24 years) is low relative to older age groups. ICC among Canadian women ages 15–19 is rare and remained constant at <0.3 cases per 100,000 women between 1970 and 2007 [25]. While minor declines in the 20–24 year and 25–29 year age groups were seen during this period, incidence rates among women ages 25–29 years were approximately four times those of women 20–24 years [25]. In BC, screening guidelines were updated in 2016 to raise the age of screen initiation from 21 to 25 years of age. Similar findings have been reported in Australia and some European countries [25].

Our findings support previous evidence indicating higher proportions of Stage IV cancers among older age groups. In Canada, stage-specific analyses found that 54.4% of ICC cases were diagnosed at Stage I, and 11.8% were diagnosed at Stage IV. Among women aged 18–39 years, 70% of cases were diagnosed at Stage I, attributed to early detection through cervical cancer screening programs. The proportion of Stage IV diagnoses was shown to increase with age, with 1 in 5 cancers diagnosed at Stage IV among women older than 55 years [19].

In BC, women can stop screening at age 69 years if they have had three negative screening tests in the past 10 years. The decision to discontinue screening is informed by the low incidence of cervical cancer in this age group and the duration of protective effects of prior screening against cervical cancer [51]. However, most people who develop cervical cancer after age 65 have not effectively participated in screening [51]. For example, a study in Denmark reported that most women aged 60 and older were diagnosed at advanced stages, with the proportion increasing with age. This was related to insufficient screening prior to exiting cervical screening, low sensitivity of colposcopy among older age groups, and exiting screening prematurely [52]. Additional approaches may be needed to ensure that older women are adequately screening prior to exiting the screening program [51,53,54]. The implementation of HPV-based screening, a more sensitive test than cervical cytology, has the potential to guide programs in exiting women from screening safely [55].

In the US, analysis by histological type and race/ethnicity found increasing rates of distant-stage SCC and AC among several age groups of non-Hispanic white women in 2011–2015, while rates remained stable among most age groups of other race/ethnicities [11]. Studies from Norway and the UK demonstrated that the majority of younger women less than 30 years were diagnosed at Stage I and had positive prognosis [56,57].

This study used population-based data from the BC Cancer Registry with at least 95% coverage for all cancer cases in the province [58]. Further, we included a long period of observation [47 years] and analysis of incidence trends by age group.

Our findings were subject to several limitations. Firstly, we did not include key sociodemographic factors. Recent studies have noted significant disparities in cervical cancer incidence and screening by population groups including women who self-report as Indigenous or a visible minority [48,59]. Secondly, trends were examined among various age groups, some of which had low numbers of cervical cancer cases which contributed to greater variability in trends. Thirdly, the age categories used for age-specific analyses are based on current screening guidelines, which may not be reflective of previous screening recommendations across the study period. Fourthly, histology was not included as an additional variable due to concerns around small numbers. Lastly, adjustment of population denominators for prior hysterectomy was not feasible in our analyses and may have led to underestimation of cervical cancer incidence rates.

## 5. Conclusions

Overall, ICC incidence rates declined in BC since 1971 but did not reach the WHO elimination target among ages 15+ years. The pace of decline varied across age groups and increased with age. Among women aged 25–69 years, attenuation in declining trends and stabilization were observed since the 1980s, particularly among the younger groups. Stage-specific differences showed an increasing ratio of late to early stage as age increased. The pathway to eliminating cervical cancer requires improving uptake of the HPV vaccine, implementations of HPV-based screening to improve screening test performance, use of innovative approaches to screening such as self-sampling to increase screening participation, and addressing inequities in access to screening and prevention services [24]. 

Canada is poised to be among the first countries worldwide to eliminate cervical cancer. This goal can be achieved by leveraging existing programs and infrastructure, and addressing key barriers to screening and vaccination. Concerted efforts are required to ensure that women of all ages have equitable access to high-quality cervical cancer prevention and care. 

## Data Availability

The data used and analyzed during the current study were obtained from the BC Cancer Registry and are not publicly available due to privacy legislation and institutional data sharing agreements. Data, however, can be requested through a data access request to BC Cancer following their processes at http://www.bccancer.bc.ca/health-professionals/professional-resources/bc-cancer-registry/request-registry-data.

## Appendix A

**Table A1:** Annual age-standardized incidence rates (ASIRs) of invasive cervical cancer for all ages 15+ years, 1971–2017.

Year	ASIR (95% CI)—2011 Canadian Population	ASIR (95% CI)—2015 World Female Population
1971	18.88 (15.86–21.90)	14.50 (12.21–16.79)
1972	15.31 (12.70–17.92)	12.40 (10.30–14.50)
1973	15.22 (12.63–17.81)	12.05 (10.01–14.09)
1974	15.69 (13.12–18.26)	12.52 (10.48–14.56)
1975	18.07 (15.38–20.76)	14.91 (12.73–17.09)
1976	19.64 (16.86–22.42)	15.97 (13.74–18.20)
1977	16.71 (14.20–19.22)	14.00 (11.94–16.06)
1978	15.92 (13.53–18.31)	13.67 (11.65–15.69)
1979	15.50 (13.17–17.83)	13.23 (11.27–15.19)
1980	14.55 (12.32–16.78)	11.92 (10.12–13.72)
1981	13.07 (10.97–15.17)	10.76 (9.05–12.47)
1982	10.32 (8.50–12.14)	8.28 (6.83–9.73)
1983	11.86 (9.94–13.78)	9.80 (8.25–11.35)
1984	9.20 (7.48–10.92)	7.50 (6.13–8.87)
1985	10.06 (8.34–11.78)	8.16 (6.79–9.53)
1986	9.63 (7.92–11.34)	7.89 (6.52–9.26)
1987	8.34 (6.79–9.89)	6.92 (5.67–8.17)
1988	9.62 (7.97–11.27)	7.79 (6.48–9.10)
1989	9.51 (7.88–11.14)	7.71 (6.42–9.00)
1990	10.10 (8.47–11.73)	8.38 (7.05–9.71)
1991	8.69 (7.20–10.18)	7.07 (5.87–8.27)
1992	9.31 (7.80–10.82)	7.67 (6.45–8.89)
1993	9.60 (8.05–11.15)	7.87 (6.64–9.10)
1994	9.48 (8.01–10.95)	7.84 (6.64–9.04)
1995	10.15 (8.64–11.66)	8.35 (7.13–9.57)
1996	8.66 (7.29–10.03)	7.12 (6.00–8.24)
1997	7.67 (6.42–8.92)	6.42 (5.38–7.46)
1998	9.69 (8.30–11.08)	8.13 (6.97–9.29)
1999	9.14 (7.77–10.51)	7.40 (6.30–8.50)
2000	6.67 (5.53–7.81)	5.76 (4.78–6.74)
2001	7.15 (5.99–8.31)	5.97 (4.99–6.95)
2002	8.22 (6.99–9.45)	6.78 (5.74–7.82)
2003	7.24 (6.08–8.40)	6.02 (5.04–7.00)
2004	6.93 (5.81–8.05)	5.82 (4.86–6.78)
2005	7.24 (6.10–8.38)	6.13 (5.15–7.11)
2006	6.77 (5.67–7.87)	5.63 (4.71–6.55)
2007	7.31 (6.19–8.43)	6.20 (5.22–7.18)
2008	7.55 (6.41–8.69)	6.22 (5.26–7.18)
2009	8.03 (6.87–9.19)	6.86 (5.84–7.88)
2010	8.11 (6.95–9.27)	6.88 (5.86–7.90)
2011	7.97 (6.81–9.13)	6.89 (5.87–7.91)
2012	7.07 (5.99–8.15)	5.99 (5.05–6.93)
2013	7.39 (6.29–8.49)	6.17 (5.23–7.11)
2014	7.70 (6.60–8.80)	6.55 (5.59–7.51)
2015	7.18 (6.12–8.24)	6.08 (5.16–7.00)
2016	6.69 (5.67–7.71)	5.72 (4.82–6.62)
2017	7.08 (6.04–8.12)	6.04 (5.14–6.94)

**Table A2:** Overall and age-specific trends in age-standardized rate of invasive cervical cancer incidence by Joinpoint Analysis, 1971–2017, using the 2011 Canadian Population Standard.

**Age Group**	**Date Range**	**APC (95% CI)**
Overall	1971–1976	2.9 (−2.8 to 8.9)
Overall	1976–1984	−7.5 (−10.6 to −4.3)
Overall	1984–2017	−1.1 (−1.4 to −0.8)
25–34 years	1971–1976	15.3 (2.1 to 30.1)
25–34 years	1976–1986	−8.0 (−11.8 to −4.1)
25–34 years	1986–2017	−0.4 (−1.1 to 0.3)
35–44 years	1971–1978	5.9 (−1.3 to 13.7)
35–44 years	1978–1982	−13.3 (−31.8 to 10.3)
35–44 years	1982–2017	0.0 (−0.5 to 0.6)
45–54 years	1971–1985	−6.3 (−9.0 to −3.4)
45–54 years	1985–2017	−0.5 (−1.3 to 0.3)
55–69 years	1971–1987	−5.2 (−6.9 to −3.4)
55–69 years	1987–2017	−1.8 (−2.6 to −1.1)
70–79 years	1971–2017	−3.2 (−3.9 to −2.6)
80+ years	1971–2017	−2.6 (−3.3 to −2.0)
**Age Group**	**Date Range**	**AAPC (95% CI)**
Overall	1971–2017	−1.8 (−2.7 to −1.0)
Overall	2008–2017	−1.1 (−1.4 to −0.8)
25–34 years	1971–2017	−0.5 (−2.1 to 1.1)
25–34 years	2008–2017	−0.4 (−1.1 to 0.3)
35–44 years	1971–2017	−0.3 (−2.6 to 2.0)
35–44 years	2008–2017	0.0 (−0.5 to 0.6)
45–54 years	1971–2017	−2.3 (−3.3 to −1.3)
45–54 years	2008–2017	−0.5 (−1.3 to 0.3)
55–69 years	1971–2017	−3.0 (−3.8 to −2.3)
55–69 years	2008–2017	−1.8 (−2.6 to −1.1)
70–79 years	1971–2017	−3.2 (−3.9 to −2.6)
70–79 years	2008–2017	−3.2 (−3.9 to −2.6)
80+ years	1971–2017	−2.6 (−3.3 to −2.0)
80+ years	2008–2017	−2.6 (−3.3 to −2.0)

APC = annual percentage change; AAPC = average annual percentage change.

**Table A3:** Overall and age-specific trends in age-standardized rate of invasive cervical cancer incidence by Joinpoint Analysis, 1971–2017, using the 2015 World Female Population Standard.

**Age Group**	**Date Range**	**APC (95% CI)**
Overall	1971–1977	2.7 (−1.5 to 7.1)
Overall	1977–1984	−8.5 (−12.4 to −4.5)
Overall	1984–2017	−0.9 (−1.3 to −0.6)
25–34 years	1971–1976	15.2 (2.3 to 29.7)
25–34 years	1976–1986	−8.1 (−11.7 to −4.2)
25–34 years	1986–2017	−0.4 (−1.1 to 0.3)
35–44 years	1971–1978	5.9 (−1.2 to 13.6)
35–44 years	1978–1982	−13.3 (−31.7 to 10.0)
35–44 years	1982–2017	0.0 (−0.5 to 0.6)
45–54 years	1971–1985	−6.3 (−9.0 to −3.4)
45–54 years	1985–2017	−0.5 (−1.3 to 0.3)
55–69 years	1971–1987	−5.2 (−6.9 to −3.4)
55–69 years	1987–2017	−1.8 (−2.6 to −1.1)
70–79 years	1971–2017	−3.2 (−3.9 to −2.6)
80+ years	1971–2017	−2.6 (−3.2 to −1.9)
**Age Group**	**Date Range**	**AAPC (95% CI)**
Overall	1971–2017	−1.7 (−2.5 to −0.8)
Overall	2008–2017	−0.9 (−1.3 to −0.6)
25–34 years	1971–2017	−0.6 (−2.1 to 1.0)
25–34 years	2008–2017	−0.4 (−1.1 to 0.3)
35–44 years	1971–2017	−0.3 (−2.6 to 2.0)
35–44 years	2008–2017	0.0 (−0.5 to 0.6)
45–54 years	1971–2017	−2.3 (−3.3 to −1.3)
45–54 years	2008–2017	−0.5 (−1.3 to 0.3)
55–69 years	1971–2017	−3.0 (−3.8 to −2.3)
70–79 years	1971–2017	−3.2 (−3.9 to −2.6)
70–79 years	2008–2017	−3.2 (−3.9 to −2.6)
80+ years	1971–2017	−2.6 (−3.2 to −1.9)
80+ years	2008–2017	−2.6 (−3.2 to −1.9)

APC = annual percentage change; AAPC = average annual percentage change.
